# Advancing Esophageal Cancer Staging and Restaging: The Role of MRI in Precision Diagnosis

**DOI:** 10.3390/cancers17081351

**Published:** 2025-04-17

**Authors:** Laura Haefliger, Pauline Chapellier, Naik Vietti Violi, Jean-Baptiste Ledoux, Styliani Mantziari, Markus Schäfer, Clarisse Dromain

**Affiliations:** 1 Department of Diagnostic and Interventional Radiology, Lausanne University Hospital and University of Lausanne, Rue du Bugnon 46, CH-1011 Lausanne, Switzerland; 2 CIBM Center for Biomedical Imaging, CH-1015 Lausanne, Switzerland; 3 Department of Surgery, Lausanne University Hospital and University of Lausanne, Rue du Bugnon 46, CH-1011 Lausanne, Switzerland

**Keywords:** esophageal cancer, MRI, TNM staging, magnetic resonance tumor regression grade

## Abstract

This review provides an in-depth analysis and comprehensive overview of recent advancements in MRI techniques for evaluating esophageal cancer (EC). It discusses the specific MRI acquisition protocols and parameters that enhance image quality and diagnostic accuracy. The review highlights MRI’s role and performance in the initial TNM staging and its potential to refine treatment strategies by improving tumor delineation and characterization. Additionally, the paper explores MRI utility in restaging after NAT, focusing on its accuracy in assessing treatment response and detecting residual or recurrent disease. Comparisons with other imaging modalities currently used—such as endoscopic ultrasound (EUS), contrast-enhanced computed tomography (CE-CT), and ^18^F-fluorodeoxyglucose (FDG) positron emission tomography/CT (PET/CT)—are included to highlight the strengths and limitations of each method. Illustrated with numerous Figures, this article proposes a novel MRI-based strategy for EC staging and restaging. It aims to integrate MRI into clinical practice by leveraging its superior soft-tissue contrast and functional imaging capabilities to enhance diagnostic precision and improve patient outcomes. Through this comprehensive evaluation, the review underscores the potential of MRI to become a cornerstone in the precision diagnosis and management of EC.

## Simple Summary

Magnetic resonance imaging (MRI) is an emerging technique for evaluating esophageal cancer and offers improved local staging, especially for advanced tumors. It also shows great potential in assessing responses to neoadjuvant treatment (NAT), similar to its use in rectal cancer. In this review, we aim to present the role of MRI in the local staging of esophageal tumors and its utility in evaluating treatment response.

## 1. Introduction

Imaging plays a central role in the diagnosis, staging, and restaging of esophageal cancer (EC). The most widely employed imaging techniques are endoscopic ultrasound (EUS), contrast-enhanced computed tomography (CE-CT), and ^18^F-fluorodeoxyglucose (FDG) positron emission tomography combined with unenhanced CT (PET/CT). Each of these techniques offer specific strengths but also come with significant limitations. EUS is particularly effective at assessing tumor invasion depth and identifying locoregional lymph nodes, although it is operator-dependent and often requires sedation or anesthesia [1]. Moreover, its accuracy may be compromised in case of stenotic tumors that limit endoscopic access.

CE-CT provides comprehensive anatomical details, and is valuable in identifying lymph nodes and metastases, particularly in the lung. However, its limited soft-tissue contrast can make it difficult to accurately define tumor margins or detect early metastases. FDG PET/CT is recommended in surgical candidates to identify distant lymph nodes and metastases that may not be detected by other modalities. However, this technique is limited by its cost, the level of radiation exposure involved, and its lower spatial resolution compared to CE-CT or EUS for local staging.

While these modalities are well established in clinical practice, their limitations—particularly in soft-tissue resolution and accuracy for local staging and restaging—highlight the need for complementary imaging technique approaches.

Magnetic resonance imaging (MRI) has emerged as a powerful imaging modality in oncology. One of its primary advantages is superior soft-tissue contrast, which facilitates accurate assessment of tumor invasion in the esophagus and surrounding structures and accurate local tumor staging [2]. Additionally, MRI can be enhanced with functional techniques such as diffusion-weighted imaging (DWI) and dynamic contrast-enhanced MRI (DCE-MRI). These techniques provide both morphological and biological information about the tumor, which can be useful for assessing treatment response and detecting early recurrence.

MRI offers significant advantages over traditional techniques: it is less operator-dependent, is non-invasive, and is more accurate than EUS in case of esophageal stenosis. Furthermore, MRI also avoids radiation exposure and is highly effective in assessing liver metastases, which is less reliable with CE-CT and FDG PET/CT. These advantages make MRI an imaging modality of choice for the precise staging and restaging of EC, with the potential to influence therapeutic decisions and improve clinical outcomes. Although not yet included in standard recommendations, the use of MRI in the initial staging of EC is expanding, and the American College of Radiology now recognizes it as a potentially appropriate method for newly diagnosed cases [3].

Despite these advantages, several challenges limit the routine use of MRI in EC. Due to the thoracic position of the esophagus, motion artifacts caused by cardiac and respiratory activity remain a well-known challenge.

Additionally, this modality typically takes longer to acquire compared to CE-CT (approximately 30 min using clinical protocols). It is, therefore, essential to optimize MRI sequences to reduce acquisition time and minimize motion and susceptibility artifacts.

The technical complexity of MRI for EC evaluation also requires not only advanced imaging equipment but also specialized expertise. Patient and tumor characteristics variabilities represent a significant challenge in image interpretation. Radiologists must be thoroughly trained in interpreting esophageal specific MRI images to ensure accurate tumor identification, staging, and treatment planning.

## 2. MRI Sequences in Esophageal Cancer Staging

The first time MRI was studied for use in cases of EC was in 1985 [4]. Since then, various MRI protocols have been developed for tumor detection, staging, or radiotherapy planning.

Acquiring high-quality MRI images of the chest, especially of the esophagus, remains technically challenging due to motion artifacts from breathing and cardiac activity. These can produce ghosting artifacts that obscure important details, particularly when evaluating the esophageal wall close to the heart and major blood vessels. Additionally, magnetic susceptibility effects—especially from air and gas in the gastrointestinal tract and airways—can distort the magnetic field and degrade the overall image quality [5]. Metallic clips from esophageal biopsy can also significantly limit the examination. Using a lower field strength (1.5T MRI) generally reduce susceptibility artifacts but may decrease the signal-to-noise ratio. Most recent studies use 3.0T MRI for better resolution [6,7].

Improving imaging begins with careful patient positioning. Proper coil placement and adjustments in patient posture can reduce magnetic susceptibility in high-risk regions such as air–tissue interfaces. Respiratory and cardiac gating helps to reduce motion artifacts, and improves image quality without significantly increasing acquisition time [8]. If cardiac gating is not available, applying a saturation band over the heart can also reduce its motion effects [9]. Some studies in China have used an antiperistaltic agent to reduce esophageal motility [4]. Additionally, using selected sequences with shorten acquisition time (e.g., short echo time sequences) or less sensitivity to magnetic susceptibility differences (e.g., spin-echo sequences) can minimize artifacts and enhance image quality [10].

Increasing the spatial resolution of MRI can improve the delineation of small anatomical structures and reduce blurring caused by artifacts. This involves using thinner slices and a smaller field of view (FOV). However, these adjustments may require longer acquisition times, which can compromise image quality if motion artifacts occur [8].

Some selected sequences are preferred in esophageal MRI due of their resistance to artifacts. The most used in a clinical protocol can be those that follow:

- T2WI multi-shot turbo spin-echo (msTSE) imaging can accurately depict the internal structure of the esophageal wall, with clear distinction between the layers and surrounding structures assisting in local staging [8,9,11]. Optimization with automatic respiratory motion compensation and diaphragm position monitoring can be applied—the end-exhalation phase is the most stable part during the respiratory cycle. Fat saturation is not routinely used, due to the lack of visibility of fat infiltration, but can help delineate tumor volume when needed [12].
- T1WI Volumetric Interpolated Breath-hold Examination (VIBE) sequence is a type of 3D Gradient Echo (GRE) used for its improved image quality, resistance to motion, and high resolution. With contrast injection, it helps to differentiate the different layers of the esophageal wall, particularly at the delayed phase [11,13,14,15]. To make it easier for patients, some free-breathing sequences have been developed. The free-breathing radial VIBE sequence and star-VIBE are fat-suppressed T1WI sequences that are less sensitive to motion.
- DWI highlights microstructural changes in tumoral tissue, aiding in the detection and extension of tumors, particularly in the esophagogastric junction [16]. There is no consensus on the optimal b-value for esophagus MRI, with values ranging from 600 to 1000. Giganti et al. used DWI with b600 at 1.5T, achieving high specificity for T staging and similar sensitivity to EUS for the N stage [16]. Chapellier et al. used b800 at 3.0T for the NAT response [17], while Shuto et al. used b1000 for lymph nodes’ assessment [18].
- ADC derived from DWI can predict the response to chemoradiotherapy (1.5 T, B0-b1000) [19]. The application of intra-voxel incoherent motion (IVIM)-DWI quantifies both water molecule diffusion and blood perfusion, with studies showing lower blood perfusion in esophagus tumors compare to the normal esophagus, probably due to a faster tumor cell proliferation than vascularization [20]. Whole-body DWI with background body signal suppression (DWIBS) has also been explored for locoregional and distant evaluation [21].

These next sequences can be an add-on for a more complete and detailed analysis:

- DCE-MRI assesses microcirculation and vascular density through perfusion measurement. It can also provide quantitive information about tumor angiogenesis and vascular permeability using some mathematic constant (K^trans^, K_ep_, and V_e_, respectively, corresponding to endothelial transfer constant, reflux rate, and fractional extravascular extracellular space volume). DCE-MRI can distinguish a normal esophagus from a malignant tumor as well as early versus advanced tumoral stages [20,22]. It is also under investigation as a means to differentiate histological types and to evaluate treatment response [15,23,24].
- Kinetic sequences adapted from cardiac MRI—steady state free precession (SSFP) in sagittal and perpendicular plans relative to the tumor—have been studied to evaluate local tumor invasiveness, taking advantage of the natural motion of thoracic structures, using cardiac gating to limit artefacts [25,26]. Cine MR sequences showed a slightly better performance in distinguishing T1–T3 from T4 with a sensitivity, specificity, and AUC of 76.5%, 83.8%, and 0.801 (0.681–0.921), and an increased in T staging confidence [26].
- T2WI TSE Dark Blood is a high-resolution sequence derived from cardiac MRI. It can be focused on the esophagus lesion, with a small FOV allowing better visibility of local invasiveness. However, this sequence is sensitive to motion and requires breath holding and cardiac gating.
- T2WI short inversion time inversion-recovery (STIR) turbo spin echo (TSE) is a quick sequence with fat saturation, and is studied for lymph node detection using their T2 signal in comparison with a normal esophagus signal to differentiate malignant from benign lymph nodes [27].
- T2* WI can reflect blood oxygenation and has the potential for quantifying tumor neoangiogenesis, which increases with higher T stages [28]. However, this sequence is highly sensitive to the presence of air, limiting its use.

An example of esophagus MRI sequences’ use in the clinical routine for EC staging is provided in Table 1.

## 3. The Role of MRI in Initial Staging of Esophageal Cancer

Accurate initial staging for EC is crucial for developing appropriate treatment strategies, particularly distinguishing between patients who are candidates for curative surgery (either upfront surgery or after NAT) and those requiring palliative care. The most widely used staging system is the TNM classification 8th edition, defined by the Union for International Cancer Control (UICC) and the American Joint Committee on Cancer (AJCC), which evaluates tumor extent (T), regional lymph node involvement (N), and distant metastasis (M) (Table 2) [29].

The two main histological types (squamous cell carcinomas and adenocarcinomas) have the same TNM but different treatment strategies. Squamous tumors typically present a lower T2 signal than a normal esophagus and often moderate contrast enhancement, with infiltrative growth patterns that can be visualized on MRI [4]. Adenocarcinomas usually appear with moderate to high T2 signal compared to a normal esophagus, and strong contrast enhancement due to their glandular structure and vascularity. However, adenocarcinomas’ appearance can vary depending on the degree of differentiation and the presence of mucinous components.

### 3.1. T Staging

While EUS remains the gold standard for assessing early-stage tumors (T1 and T2), MRI is of interest in evaluating locally advanced EC and should be used in cases of severe or complete stenosis.

**Figure 1:**
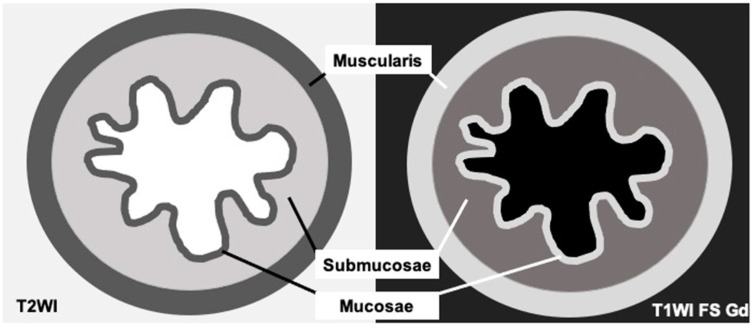
Scheme of esophagus wall appearance on MRI. Sequence T2WI: the mucosa is thin and hypointense, the submucosa is the most visible layer and is hyperintense, while the muscularis propria is the third visible layer and appears hypointense. Sequence T1WI FS at 3 min post-gadolinium injection: the mucosa is thin and hyperintense, the submucosa is hypointense, and the muscularis propria is hyperintense due to enhancement. WI: weighted imaging; FS: fat sat; and Gd: gadolinium.

MRI is highly effective at distinguishing soft-tissue contrast, making it useful in differentiating between esophagus layers, tumor, and adjacent structures [2,7]. In ex vivo analysis, up to eight layers have been visualized, with an excellent correlation between MRI and histopathological analysis [30]. Due to spatial resolution, in in vivo settings, only three layers can be distinguished: mucosa, submucosa, and muscularis propria (Figure 1) [1,8,31]. While the invasion of mucosa and sub-mucosa is hardly distinguishable on MRI, the invasion of muscularis propria and the adjacent organ can be effectively detected.

Sensitivity and specificity for T staging with MRI vary significantly, ranging from 33% to 100% and 55% to 100%, respectively, with higher values reported for locally advanced tumors (Table 3). In a meta-analysis from Lee et al. in 2021, pooled sensitivity and specificity for early-stage (T1–T2) versus advanced-stage (T3–4) tumors were, respectively, 92 and 86%, with specificities of 67 and 86% [7].

Compared to EUS, MRI has shown similar sensitivity and accuracy for lower stage tumors (2 readers, 90.5 and 100% vs. 100%) and even higher accuracy for advanced stages (2 readers, 81.8% and 90.0% vs. 68%) [13]. The overall accuracy compared to histopathological staging is around 63.2% [36].

**Figure 2:**
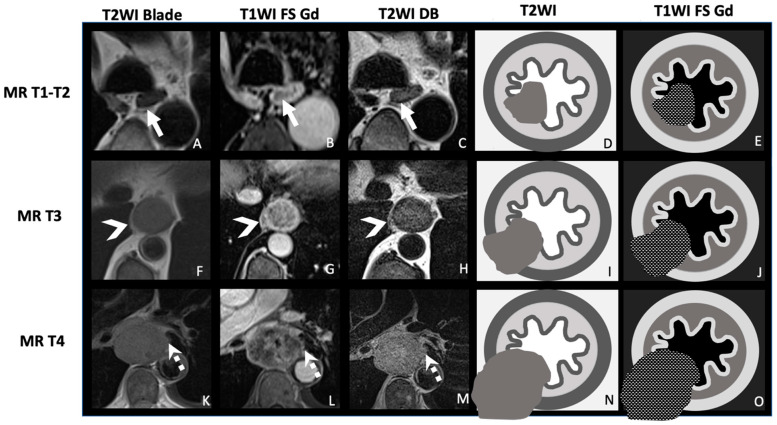
Illustrated scheme of MRI for T staging. This illustration uses T2WI BLADE sequence (**A**,**F**,**K**), T1WI FS Gd (**B**,**G**,**L**), and an optional T2WI DB (**C**,**H**,**M**), along with illustrative diagrams (**D**,**E**,**I**,**J**,**N**,**O**). T1 and T2 lesions (**A**–**E**) are not dissociable on MRI due to the in vivo resolution. The white arrows indicate a MR-T1/T2 lesion in the right side of the esophagus without invasion of the muscularis propria (**A**–**C**). MR-T2 lesions show intermediate signal intensity on T2WI and on T1WI, usually smaller than the submucosae. T3 lesions (**F**–**J**) are often circumferential with infiltration of the adjacent fat, sometimes accompanied by tumoral deposits. It can be associated with irregularity in the muscularis propria margin. Arrowheads highlight periesophageal fat infiltration in a circumferential MR-T3 lesion. T4 lesions (**K**–**O**) are locally invasive, with extension into adjacent organs. There is a loss of the fat plane between the esophagus and the adjacent structure. In this example, the lesion is circumferential, with invasion of the adjacent left pleura, which is irregular, with spiculated contours ff(dashed arrow), classifying it MR-T4a (**K**–**M**). Images acquired on a 3 T-scanner (Magnetom PrismaFit, Siemens Healthcare). WI: weighted imaging; TSE: turbo spin echo; and DB: dark blood.

**Figure 3:**
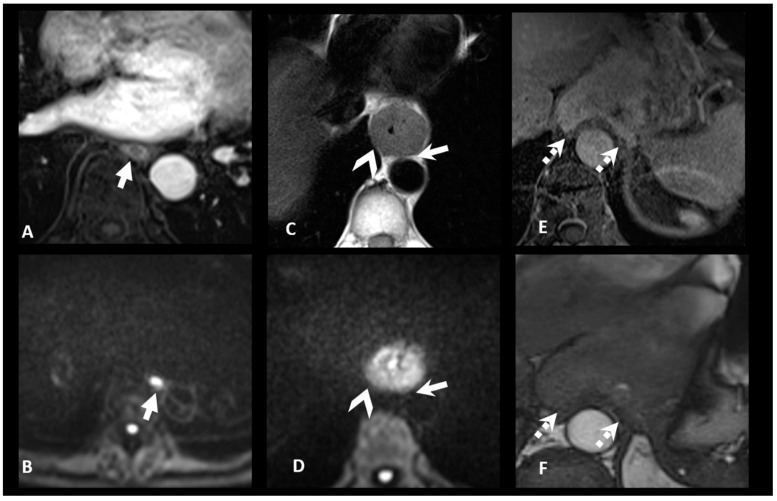
Contribution of MRI for T staging. The transverse MRI images in T1WI FS GD (**A**) and DWI (**B**) show a small tumor in the middle third of the esophagus without invasion of the adjacent fat, classified as MR-T1/2. The T2WI and DWI transverse MRI images (**C**,**D**) show a tumor in contact with right pleura (arrowhead) and focally with aorta (arrow), without invasion, classifying this tumor as MR-T3. The transverse MRI images in T1WI FS GD show a large tumor of gastroesophageal junction Siewert 3 (**E**,**F**) invading diaphragmatic pillars (dashed arrow) with loss of peripheric hyposignal on kinetic SSFP sequences, classifying the tumor as MR-T4a. Images acquired on a 3 T-scanner (Magnetom PrismaFit, Siemens Healthcare). WI: weighted imaging; DWI: diffusion-weighted imaging; FS GD: fat sat with gadolinium; and SSFP: steady state free precession.

**Figure 4:**
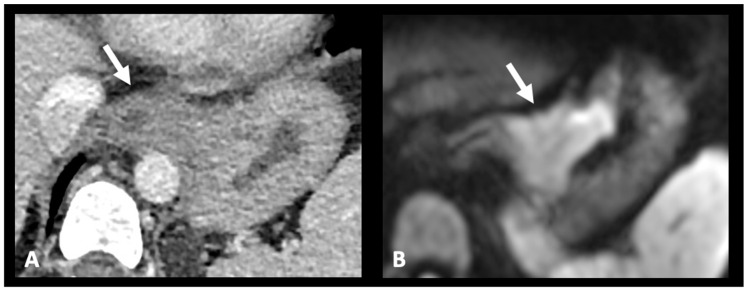
Contribution of MRI in assessing tumor location and extension. The transverse CE-CT image (**A**) shows a tumor at the gastroesophageal junction with its epicenter located above the cardia, classified as CT-Sievert 1. The MRI image on DWI (**B**) shows significant diffusion restriction at the level of the lesser gastric curvature, reclassifying this gastroesophageal junction tumor as a Sievert 3 tumor. DWI: diffusion-weighted imaging and CE-CT: contrast-enhanced computed tomography.

Studies have suggested that MRI, especially when using T2WI and DWI, can more clearly differentiate between early (T1–T2) and advanced (T3–T4) disease stages [37]. T2WI is particularly of interest for identifying peri-esophageal fat invasion (T3) [26]. The use of fat-suppressed T1WI sequences acquired in the delayed post-contrast phase can help in detecting the early-stage disease (Figure 2, Figure 3 and Figure 4).

Accurately assessing thoracic aorta and tracheobronchial invasion is probably the most challenging, even with advanced MR sequences and expert radiologists. Zhao et al. compared the diagnostic performance of 3.0 T MRI and CE-CT in detecting such invasions in 70 patients with EC. Their results showed that 3.0 T MRI outperformed CE-CT in diagnosing these invasions, although the results varied depending on tumor location and reader experience. Sensitivity and specificity ranged from 85.7% to 92.3% and 90.1% to 96.4%, respectively [38]. These inconsistencies may arise from tumor-induced compression effects, leading to false positives due to surrounding inflammation and oedema. Moreover, distinguishing the esophageal muscularis from adjacent organs is more difficult in patients with low body fat, increasing the risk of over- or underestimation.

### 3.2. N Staging

During the EUS procedure, fine-needle biopsy of suspicious lymph nodes can be performed, providing both imaging and tissue sampling, which increases the accuracy of N staging. EUS has a sensitivity ranging from 59.5% to 97.2% and a specificity between 40% and 100% [39].

FDG PET/CT detects metabolically active lymph nodes, but has a moderate sensitivity for loco-regional detection due to the proximity of tumor and regional lymph nodes (pooled sensitivity and specificity with a PET of 51% and 84%, respectively) [40].

Unlike EUS, MRI allows the evaluation of lymph node chains over a broad anatomical region, covering the entire thorax (including the root of the neck) and the abdomen.

**Figure 5:**
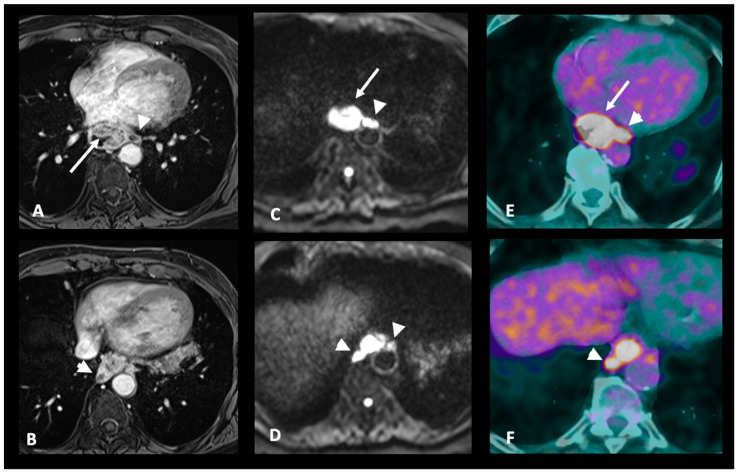
A 69-year-old patient presenting with an endoscopically unpassable tumor. The MRI images in T1WI FS GD (**A**,**B**) and DWI (**C**,**D**) show a large tumor in the lower third of the esophagus invading the periesophageal fat (white arrows). Multiple necrotic periesophageal lymph nodes are also present (arrowheads), classifying the tumor as MR-T3N2. The FDG PET/CT images (**E**,**F**) show intense tracer uptake at the esophageal tumor and periesophageal lymph nodes, confirming their tumoral nature. MRI Images acquired on a 3 T-scanner (Magnetom PrismaFit, Siemens Healthcare). WI: weighted imaging; DWI: diffusion-weighted imaging; and FS GD: fat sat with gadolinium.

MRI identifies lymph node metastasis based on size, morphology, and the presence of necrosis or enhancement patterns with a sensitivity reported between 64 and 82% and specificity between 75 and 82% [41] (Figure 5). Using DWI (ADC <1.2 mm/s^2^ (b0-b1000)) instead of FDG PET/CT slightly increased sensitivity (68 vs. 62% in the Shuto et al. study), particularly in the case of upper chest lymph nodes and when there is a large cancer nest area [18].

Extending the size definition in MRI by considering a pathological lymph node with 5 mm of short axis instead of 10 mm increases sensitivity, specificity, and accuracy (respectively, 100, 95, and 96%) [42]. The signal on T2WI is also considered, using higher signal intensity compared to the signal of the non tumoral esophagus, as a sign of metastatic lymph nodes [27].

### 3.3. M Staging

FDG PET/CT is the gold standard for detecting distant metastases due to its ability to highlight metabolic activity; however, it has limitations in terms of detecting liver metastases. CE-CT also performs well for M staging, especially in the lungs and the liver, but is less sensitive than FDG PET/CT for detecting bone metastases, distant lymph nodes metastases, or an unexpected site [43]. MRI is the gold standard for liver metastasis detection—its sensitivity ranges from 76 to 86% and its specificity from 94 to 99% [44]. For this reason, an esophageal MRI should always include dedicated liver sequences.

Whole-body MRI is an interesting alternative for evaluating metastatic disease. Encouraging results have been presented in colorectal cancer with the Streamline C trial, showing similar accuracy for N and M staging compared to FDG PET/CT, while reducing the number of tests and the overall staging time [33,45,46]. Currently, the use of whole-body MRI remains limited to clinical trials, but it could become an alternative to FDG PET/CT for selected patients for assessing both the local and distant spread in a single examination—though the examination time could be long.

## 4. The Role of MRI in Restaging and Monitoring Treatment Response

The vast majority of patients diagnosed with EC are diagnosed in locally advanced stages of the disease (cT3 and/or cN+, M0) [47]. For these patients, neoadjuvant chemoradiation therapy (CRT) or chemotherapy (CT) is the standard treatment, followed by in-bloc oncologic esophagectomy [47]. Current neoadjuvant CRT regimens are increasingly efficient in achieving complete clinical response (cCR), as reported for 49% of squamous cell and 12–23% of adenocarcinoma patients [48,49,50]. Thus, a definitive CRT with a watch and wait strategy instead of systematic surgical resection is increasingly considered, even in the more radiation-resistant adenocarcinoma patients. Readily available, reliable, and reproducible restaging methods are key for the successful management of these patients, and to limit the risk of underestimating the presence of residual disease. Currently a rigorous multi-modal protocol of restaging after CRT has been assessed in the pre-SANO trial, with a combination of EUS, bite-on-bite biopsies, and fine-needle aspiration of suspicious lymph nodes and FDG PET/CT [51]. Although the overall diagnostic accuracy of this model was considered as “adequate”, false negative results were observed in 18–82% of patients after regular biopsies, 0–41% of patients after bite-on-bite biopsies, and 31–55% of patients after EUS [51].

While these restaging methods are invasive, MRI could be key in the restaging assessment on the same way it is used currently in rectal cancer restaging. It could help to select patient requiring EUS and targeted biopsies to reduce false negative rates. MRI evaluation after NAT can be particularly challenging as the tumors may shrink, become necrotic, or exhibit fibrotic changes. These alterations can make it difficult to distinguish between residual viable tumor tissue and treatment-related changes such as fibrosis or necrosis [17].

Currently, the restaging after NAT is based on three different strategies [52]:

(1) no assessable disease, so patients might benefit from an active surveillance approach or esophagectomy;
(2) persistent locoregional disease with no distant metastasis, so patients might require subsequent surgery;
(3) evidence of distant spread, so patients should receive systemic treatment.

### 4.1. T Restaging

The currently recommended CE-CT and EUS imaging modalities appear to be insufficiently accurate to allow a distinction to be made between the residual tumor and the local changes associated with CRT, and this can result in ycT overstating [53,54]. Furthermore, EUS lacks in precision in delineating the esophageal layers after NAT, given the induced architectural distortion [55].

The accuracy of FDG PET/CT for detecting complete response remains low, even after 12 weeks post CRT, when the radiation-induced inflammation has receded [56]. De Gouw et al. concluded that CE-CT, FDG PET/CT, and EUS are unreliable for identifying complete responders (pCR), with pooled sensitivities of CE-CT, FDG PET/CT, EUS of 35%, 62%, and 1% and pooled specificities of 83%, 73%, and 99%, respectively [57]. Thus, in order to adhere to a watchful wait strategy, an alternative modality is required, and MRI could be key to predict CR during neoadjuvant CRT.

A combination of FDG PET/CT and DWI-MRI has a better accuracy than FDG PET/CT alone in the identification of the response to neoadjuvant CRT of a primary tumor. In a study of 54 patients with squamous cell carcinoma, a combination of the ADC at mid-treatment timing and FDG PET/CT measurement of total lesion glycolysis (TLG) at post-treatment timing, had the highest AUC of 0.914, with high sensitivity and specificity to predict the pathological response of a primary tumor (90.00% and 86.36%, respectively) [58].

A NAT preoperative assessment using MRI, mainly based on T2WI, has great sensitivity to detect a residual tumor, ranging from 90 to 100%, reflecting a low chance of missing a residual tumor [59]. However, the specificity is low, ranging from 8 to 25%, causing a great risk of overstating, and, consequently, overtreating [59]. Promising results have recently been reported when assessing a complete response prediction using an early regression index (ERI) in a recent small Italian series. In this study, a measure of tumor volume on T2WI and a calculation of ERI was performed during the MRI pre-treatment of 25 patients, at mid-radiotherapy CRT, and after treatment. ERImid showed better performance than the evaluation post-treatment (AUC: 0.78, *p* = 0.014), with a sensitivity of 88% and a specificity of 64%; A two-variable logistic model combining ERImid and volume pre-treatment improved performances (AUC: 0.93, *p* < 0.0001), with high sensitivity and specificity (100% and 82%, respectively) [60].

The incorporation of DWI and ADC sequences has significantly enhanced the evaluation of NAT responses, with the presence of a high signal on DWI within the tumor bed (with a corresponding low ADC value) indicating a high cellularity residual tumor. A study by Borggreve et al. was conducted to determine the optimal timing of DWI MRI for predicting pCR in neoadjuvant CRT. The relative change in the tumor apparent diffusion coefficient (ΔADC (%)) during the first two weeks of nCRT appeared to be the most predictive for pCR. During this period, the model was able to discriminate between pCR and non-pCR in 87% [61].

In the Vollenbrock et al. study, the addition of images obtained by functional DW-MRI to the anatomical T2WI-MRI images did not significantly influence the overall diagnostic performance, but had a positive impact on the specificity (ranging from 42% to 50%) and interobserver agreement [59].

Some authors found that the ADC value tends to be a good marker to predict the patient’s prognosis and treatment response, with responders having a lower baseline and higher post-treatment ADC values compared to non-responders [19,62,63]. In a recent meta-analysis including 236 patients treated by CRT, the pooled sensitivity and specificity of MRI for predicting early response to CRT was 93% (95% CI: 77–98%) and 85% (72–93%) for the Δ ADC, respectively; and 75% and 90% for the post-treatment ADC, respectively [64].

DCE-MRI has also been shown to be a promising tool for restaging, by assessing perfusion changes after NAT. In a study of 26 patients, Heethuis et al. showed that decreased perfusion parameters correlated with a better pathological response. The change in tumor area-under-the concentration-time-curve had a sensitivity of 83%, specificity of 88%, PPV of 71%, and a NPV of 93% for predicting pCR [65].

**Figure 6:**
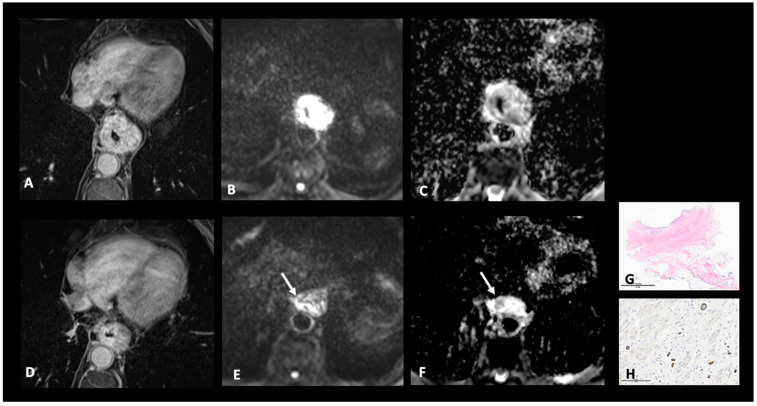
Contribution of MRI for restaging after NAT. Pre-treatment MR images, including T1WI FS GD (**A**), DWI b = 800 (**B**), and the corresponding ADC map (**C**), show a tumor located in the lower third of the esophagus, classified as cT3N2. After NAT, the T1WI FS GD MR image (**D**) shows a moderate reduction in size, measured at −33%. Tumor is restaged as yMRT3N1. However, only a small portion (<50%) of the residual mass (arrow) exhibits high signal intensity on DWI (**E**) and low signal intensity on the ADC map (**F**). The response is classified as MR-TRG 3 and 2, according to Mandard and Becker scores, respectively. Histological analysis of the surgical specimen, HE stain at magnification × 0.3 (**G**) and CKAE1/AE3 immunostaining at magnification × 20 (**H**), reveals an ulcerated fibrous scar with few viable atypical cells in the tumor bed, classified as pTRG 3 and 2 according to Mandard and Becker scores, respectively. NAT: neoadjuvant treatment; WI: weighted imaging; DWI: diffusion-weighted imaging; and FS GD: fat sat with gadolinium.

Recent articles have also presented a new esophageal MR tumor regression grade (TRG), based on the pre-existing histological TRG [66] and the commonly used MR-TRG for rectal cancer [67]. This tool is a scoring system based on the DWI and T2WI sequences, which evaluates the tumor response to NAT according to the relative amount of remaining viable tumor tissue and induced fibrosis [17,68]. Chapellier et al. assessed the concordance between histological and MR-TRG scores in 28 patients and found a good correlation when grouping grade 1-2 for the Mandard score and 1a-1b for the Becker suggesting that MR-TGR could be used as a surrogate marker for complete and near-complete pathological response [17] (Figure 6).

Finally, Jinrong Qu et al. [68] introduced a quantitative method derived from traditional RECIST criteria (qRECIST) with the addition of tumor size, T2 tumor signal, ADC value, and enhancement pattern. They found that delta RECIST built using the change in parameters between post-NAT and pre-NAT MRI measurements achieved the highest accuracy (97.8%) in distinguishing responders from non-responders.

### 4.2. N and M Restaging

EUS and CE-CT do not seem adequate for the regional lymph nodes staging after treatment [29,57]. In the M Van Pliet study, pooled sensitivities of EUS and CE-CT for regional lymph node assessment after CRT were 80% and 50% with specificities of 79% and 83%, respectively [69]. This moderated sensitivity can partly be explained by the fact that short axis measurement remains the unique imaging criteria for lymph node evaluation.

In the future, further attention should be paid to the assessment of metastatic lymph nodes’ response to treatment because a complete primary tumor response may, however, be accompanied by residual malignancy in the locoregional lymph nodes.

FDG PET/CT is commonly used for restaging, especially for patients who are candidates for surgery. It has an established role in detecting interval distant metastases occurrence, appearing in 8–10% of patients after CRT [56,70].

There are few studies assessing the MRI value for the early detection of tumor recurrence or resistance to treatment. One study compared CE-CT with whole-body T2WI MRI and concluded the superiority of CT in detecting lung metastasis, while MRI was superior in the evaluation of esophageal wall thickening and bone metastasis [71]. Another study found no significant difference in accuracy between DWI and FDG PET/CT in detecting locoregional lymph node recurrence for patients with EC [72].

However MRI, particularly with DWI and functional imaging techniques, can detect residual tumor tissue or early recurrence before conventional imaging modalities [73], as is the case for rectal cancer.

As in initial staging, whole-body MRI could be used as a surrogate for FDG PET/CT for recurrence detection. It is a valuable encouraging method for oncologic follow-up, reducing radiation exposure with excellent delineation between normal and pathological tissues [46].

## 5. Future Directions and Research Priorities

Researchers are exploring the use of MRI not only as a standalone tool but also in combination with traditional imaging modalities to develop multi-modal imaging approaches.

Radiomics and artificial intelligence (AI) are being integrated into MRI analysis to improve accuracy in differentiating benign from malignant lymph nodes and better assessing tumor spread. These developments could lead to a more prominent role for MRI in the future.

The development of AI-based sequences, allowing faster acquisition and noise reduction, is particularly beneficial for esophagus MRI with its movements limitations, and can participate to the diagnostic accuracy improvement [74]. AI algorithms, especially deep learning models, have been employed to automatically segment esophageal tumors and extract radiomic features from MRI scans. With a direct clinical application, Wang et al. proposed a AI-based model of automatic segmentation that can be apply for feature extraction but also for radiotherapy planning [4].

Several studies have shown that radiomics features extracted from MRI, particularly from DWI sequences, can improve the accuracy of T and N stages: a recent meta-analysis evidences the potential added value of radiomics for the prediction of lymph node metastasis in EC with a pooled sensitivity and specificity of 72% and 76%, respectively [75].

Radiomics data extraction based on pre-treatment MRI demonstrated the ability to predict a patient’s response to neoadjuvant CRT. Radiomics models built from MRI data have been shown to predict long-term outcomes, such as overall survival and progression-free survival. By analyzing the tumor’s radiomic signature, AI models can identify high-risk patients who may benefit from more aggressive treatments or closer follow-up. DCE and DWI have been shown to have encouraging effects in predicting tumor response to neoadjuvant CRT and patient survival [65,76].

## 6. Integrating Radiomics with Genomics and Clinical Data

Even if the literature is scarce when considering radiogenomic models for EC including MRI, there is a promise in predictive models that incorporates MRI-based radiomics to genomics and clinical factors, to create comprehensive predictive models [77].

Several clinical trials are underway to evaluate the role of MRI in the initial staging of EC, with a particular focus on combining it with other imaging techniques such as CE-CT, FDG PET/CT, and EUS. Some key trials include:

- The use of MRI to assess treatment response is under investigation in multiple centers. The RELAY study (United States, 2020–2025, NCT04188535) investigates the feasibility and utility of using serial MRI to assess treatment response during and after radiation therapy for patients with advanced EC but also with glioblastoma, prostate cancer, vulvar cancer, or pediatric glioma. A Chinese study (2016–2025, NCT02988921) aims to explore the value of MRI for the prediction of tumor response to CRT and accurate target volume delineation as compared to CT simulation for patients with unresectable or potentially resectable EC.
- Personalized patient management is under investigation in the PIONEER study (United States, 2021–2025, NCT04846309) where MRI and ^18^F-fluoromisonidazole (FMISO)-PET are being used to evaluate tumor hypoxia. Patient with tumor hypoxia will receive a higher dose of radiation therapy while those without hypoxic tumor will be treated with the standard-of-care radiation regimen.
- PET/MRI is being investigated in multiple clinical trials: the Escape study (Italy, 2020–2025, NCT04359732) aims to investigate the potential role of PET/MRI as a predictor of response to NAT in EC. The FAZA study (Canada, 2020–2024, NCT04560036) is currently investigating the use of PET/MRI with a specific radiotracer, ^18^F-Fluoroazomycin Arabinoside (FAZA), before and after standard of care platinum-based chemotherapy for patients with metastasized EC, with the goal of improving the treatment response assessment.
- MRI, as a guide for radiotherapy, is currently being investigated in a German study (2018–2027, NCT04172753) using the 1.5 T hybrid magnetic resonance imaging—linear accelerator system (MR-Linac) to delivered radiotherapy with high precision for EC.
- Ongoing research is exploring how combining MRI with CT can improve overall staging accuracy and to assess the response after NAT (China, 2016–2025, NCT02988921).

## 7. Clinical Implications: How Advancements in MRI and Multi-Modal Approaches Can Improve Patient Management

A combination of different modalities may improve overall diagnostic accuracy, lowering staging errors that may compromise management and prognosis of patients in an era where watchful waiting in cases of EC with cCR is tending to gain popularity.

As endoscopic assessment of residual disease may be particularly difficult in the presence of a stenotic lesion, esophagitis, or post-CRT fibrosis, and non-invasive imaging modalities may have better performance as they can be performed in case of tumor stenosis.

While each imaging modality has its strengths, combining them into a multi-modal approach offers the potential to overcome the limitations of each individual technique. As presented in this review, a combination of MRI with conventional imaging techniques can enhance the overall accuracy of EC staging.

Combining EUS with MRI can be of interest in T staging to perform personalized patient management by using EUS for a small tumor while keeping MRI for a completely stenotic tumor or a locally advanced tumor, allowing high performance for the assessment of adjacent structures’ invasion. The combination of both exams can be reserved for patients with difficult-to-assess tumors, by complementing the high-resolution images of EUS with MRI’s superior soft-tissue contrast.

The combination of CT or FDG PET/CT and MRI is of interest as they are non-invasive and relatively available techniques. MRI can focus on the tumor T stage, local lymph nodes, and liver metastasis assessment. CT or FDG/PET-CT have to be used for distant pathological lymph nodes’ assessment and lung, bone, or other distant metastatic sites.

The combination of MRI for liver metastasis and PET/CT for other metastatic sites is probably currently the most sensitive combination for metastasis detection and should be used for patients considered for surgery to rule out metastasis.

In the re-evaluation after NAT, MRI’s sensitivity in differentiating between fibrosis and viable tumor tissue can be valuable, helping to assess treatment response before surgical resection. Early detection of treatment response can help in modifying or intensifying treatment regimens. For instance, patients who show a good response to MRI may be candidates for less extensive surgery, while those with a poor response might require more aggressive treatment.

In the future, functional MRI could help personalize treatment plans by identifying patients who are likely to benefit from alternative therapeutic strategies based on tumor biology. MRI could also play a role in personalized strategies, assessing the efficacy of NAT or to monitor for disease recurrence following surgery.

## 8. Conclusions

MRI is a valuable and reliable tool for the staging and restaging of EC. Compared to traditional techniques, its ability to provide better soft contrast, tissue differentiation, and detailed and more precise tumor characterization can become crucial for the clinical decision process in patient management. These advantages support more precise staging and improved assessment of treatment response.

As research continues, AI and radiomics may also play a role in enhancing MRI’s capabilities by automatically segmenting tumors and lymph nodes, potentially improving diagnostic performance.

By enabling more accurate and less invasive tumor staging and treatment response assessment, MRI can contribute to more informed therapeutic decisions, reducing the use of unnecessary treatments and improving patient outcomes.

## Figures and Tables

**Table 1:** Clinical MRI sequences’ parameters on 3.0-T-scanner (Magnetom PrismaFit, Siemens Healthcare).

	T2WI TSE Blade	T2WI TSE Blade	DWI Chest	DWI Liver	T1WI Dynamic VIBE Dixon	OPTIONAL T2WI TSE DB
Plane	Axial	Sagittal	Axial	Axial	Axial	Axial
Volume	Chest and liver 2 boxes	Chest	Chest	Liver	Chest and liver 2 boxes	Perpendicular to Lesion
Repetition Time TR (ms)	2290	2020	3500	2500	4.5	550 ^(1)^
Echo Time TE (ms)	68	89	59	59	1.35–2.58	69
Slice Thickness (mm)	5	3	5	5	2.3	3
Field of View (mm)	371 × 371	400 × 400	380 × 261	380 × 261	350 × 273	230 × 187
Phase Encoding	x (Radial)	x (Radial)	A > P	A > P	A > P	A > P
Matrix Size	320	320	134 × 134	134 × 134	320 × 240	256 × 230
Voxel Size (mm)	1.2 × 1.2 × 5	1.3 × 1.3 × 3	1.4 × 1.4 × 5 (interpolated)	1.4 × 1.4 × 5 (interpolated)	1.1 × 1.1 × 2.3	0.9 × 0.9 × 3
Number of Slices	45	30	40	28	88	25
Distance Factor (mm)	1	0.3	1	1	0.46	0,3
Flip Angle (degree)	100	120	x	x	9	180
Acceleration Factors	Grappa 3	Grappa 2	SMS 4	SMS 4	Caipirinha 4	Grappa 2
Turbo Factors (or EPI Factor)	35	31	92	92	x	17
Bandwidth (Hz/Px)	781	1563	2332	2332	1040	849
Nex	1	1	1	1	1	1
Acquisition Time (min)	1 min 23 s	1 min 51 s	1 min 43 s	1 min 14 s	18 sec/phase	8 min 34 s ^(1)^
Number of Apnea	4	4	FB	FB	4	13–25 ^(1)^

(1) Heart rate dependent. WI: weighted imaging; TSE: turbo spin echo; DWI: diffusion-weighted imaging; VIBE: volume interpolated breath-hold examination; TR: repetition time; TE: time to echo; EPI: echo planar imaging; Hz/Px: hertz/pixel; and FB: free breathing. Two 16-channel body array coils and a 32-channel spine coil (Siemens Healthcare) were employed.

**Table 2:** TNM, according to AJCC/UICC 8th edition, adapted to MRI.

TNM Histo-Pathologi Definition (According to AJCC/UICC 8th Edition)	MRI TNM Staging
**T1**: Tumor invades the submucosa	**MR-T1-T2**: Tumor signal intensity is confined to esophageal wall without extension to peri-esophagus fat
**T2**: Tumor invades the muscularis propria	
**T3**: Tumor invades adventitia	**MR-T3**: Tumor invades the peri-esophagus fat tissue
**T4**: Tumor invades adjacent organ T4a resectable organs: pleura, pericardium, azygos vein, diaphragm or peritoneum T4b unresectable organs: aorta, vertebral body or trachea	**MR-T4**: Tumor invades adjacent organs T4a resectable organs: pleura, pericardium, azygos vein, diaphragm or peritoneum T4b unresectable organs: aorta, vertebral body or trachea
N+: Regional lymph nodes metastasis	**MRI-N+**: Round, Intermediate to high T2 signal, heterogeneous T1 Gd, >5 mm small axe
M+: Distant metastases	**MRI-M+**: Distant lymph node (sus-clavicular e.g.), liver metastases or other organ metastases

**Table 3:** Selected studies with sensitivity and specificity for MRI TNM staging.

First Author, Year	No. of Patients	MRI	Sequence	T Staging		N Staging		M Staging	
				Sens %	Spe %	Sens %	Spe %	Sens %	Spe %
Ling-Fei Wu et al., 2003 [32]	86	0.15 T	T1WI and T2WI	40–55	55–63	NA	NA	NA	NA
Sakurada et al., 2009 [21]	24	1.5 T	DWIBS (b0-1000), T2WI	33–100	Not provided	>5 mm 77.8	55.6	NA	NA
Malik et al., 2015 [33]	49	3 T	DWIBS (b0-1000), T2WI	59–98	92–100	30	100		
Lei et al., 2015 [20]	30	3 T	IVIM-DWI (b0-1500), DCE MRI	90	95	NA	NA	67	91
Wei et al., 2016 [30]	61	3 T	T2WI	71–100	98–100	NA	NA	NA	NA
Giganti et al., 2016 [16]	18	1.5 T	T2WI, DWI (b0-600)	67	83	>10 mm 100	57	NA	NA
Qu et al., 2018 [13]	43	3 T	T1WI-CE-(VIBE)	73–100	85–100	NA	NA	NA	NA
Wu et al., 2018 [34]	60	3 T	T2WI, CE-T1WI, DWI (b-values 0-800)	84–100	100	NA	NA	NA	NA
Chen et al., 2019 [22]	42	3 T	DCE-MRI	86.2 (V_e_)	61.5	77.8 (K^ep^)	59.7	NA	NA
Guo et al., 2020 [11]	74	3 T	T2WI msTSE, DWI (b0-700), T1WI 3D GRE	96–98	93	NA	NA	NA	NA
Shuto et al., 2020 [18]	76	1.5 T	DWI (b0-1000)	NA	NA	>10 mm; ADC < 1.2 s/mm^2^ 67	98	NA	NA
Wang et al., 2022 [35]	35	3 T	T2IW, DWI (b0-800)	85.7	77.1	47.8	91.5	NA	NA
Haefliger et al., 2023 [26]	53	3 T	T2WI, DWI (b0-800), T1WI-CE, Cine	95.8	75	NA	NA	NA	NA

WI: weighted imaging; DWI: diffusion-weighted imaging; DWIBS: diffusion-weighted imaging with background suppression; IVIM-DWI: intra-voxel incoherent motion-diffusion-weighted imaging; DCE: dynamic contrast-enhanced magnetic resonance imaging; msTSE: multi-shot turbo spin-echo sequence; and 3D-GRE:3D gradient-echo-based sequence.

## Abbreviations

ADC	Apparent diffusion coefficient
AJCC	American Joint Committee on Cancer
CT	Computed tomography
CE-CT	Contrast-enhanced computed tomography
CR	Complete clinical response
nCR	near-complete response
CRT	Chemoradiotherapy
DB	Dark blood
DCE	Dynamic contrast-enhanced magnetic resonance imaging
DWI	Diffusion-weighted imaging
EC	Esophageal cancer
EUS	Endoscopic ultrasound
FB	Free breathing
FDG PET/CT	Positron emission tomography with [^18^F]-fluorodexoxyglucose computed tomography
GRE	Gradient echo
IVIM-DWI	intra-voxel incoherent motion-diffusion-weighted imaging
MRI	Magnetic resonance imaging
MR-TRG	Magnetic resonance tumor regression grade
NAT	Neoadjuvant treatment
pCR	pathological complete response
pTRG	pathologic tumor regression grade
RECIST:	Response evaluation criteria in solid tumors
TE	Time to echo
TR	Time repetition
Ms TSE	Multi-shot turbo spin echo
TNM	Tumor, node, metastasis
SSFP	steady state free precession
STIR	short inversion time inversion recovery
UICC	Union for International Cancer Control
VIBE	Volume interpolated breath-hold examination
WI	Weighted imaging
ypTNM	pathological classification of malignant tumors after neoadjuvant treatment
